# Using intervention mapping to develop ‘ROSE’: an intervention to support peer workers in overdose response settings

**DOI:** 10.1186/s12913-021-07241-2

**Published:** 2021-11-27

**Authors:** Zahra Mamdani, Sophie McKenzie, Fred Cameron, Mike Knott, Jennifer Conway-Brown, Tracy Scott, Jane A. Buxton, Bernie Pauly

**Affiliations:** 1 BC Centre for Disease Control, 655 West 12th Avenue, Vancouver, BC V5Z 4R4 Canada; 2 SOLID Outreach Society, 1056 N Park St, Victoria, BC V8T 1C6 Canada; 3 RainCity Housing, 616 Powell St, Vancouver, BC V6A 1H4 Canada; 4 School of Population and Public Health, University of British Columbia, 2206 East Mall, Vancouver, BC V6T 1Z3 Canada; 5 Canadian Institute for Substance Use Research, University of Victoria, Box 1700 STN CSC, Victoria, BC Canada

**Keywords:** Peer workers, overdose response, intervention mapping, organization, stigma, lived/living experience, substance use

## Abstract

**Background:**

Peer workers (those with lived/living experience of substance use working in overdose response settings) are at the forefront of overdose response initiatives in British Columbia (BC). Working in these settings can be stressful, with lasting social, mental and emotional impacts. Peer workers have also been disproportionately burdened by the current dual public health crises characterized by the onset of the COVID-19 pandemic and rise in illicit drug overdose deaths. It is therefore critical to develop supports tailored specifically to their realities.

**Methods:**

We used the six steps outlined in the Intervention Mapping (IM) framework to identify needs of peer workers and design an intervention model to support peer workers in overdose response settings.

**Results:**

Eight peer-led focus groups were conducted in community settings to identify peer workers’ needs and transcripts were analyzed using interpretive description. The strategies within the intervention model were informed by organizational development theory as well as by lived/living experience of peer workers. The support needs identified by peer workers were categorized into three key themes and these formed the basis of an intervention model titled ‘ROSE’; R stands for Recognition of peer work, O for Organizational support, S for Skill development and E for Everyone. The ROSE model aims to facilitate cultural changes within organizations, leading towards more equitable and just workplaces for peer workers. This, in turn, has the potential for positive socio-ecological impact.

**Conclusions:**

Centering lived/living experience in the intervention mapping process led us to develop a framework for supporting peer workers in BC. The ROSE model can be used as a baseline for other organizations employing peer workers.

**Supplementary Information:**

The online version contains supplementary material available at 10.1186/s12913-021-07241-2.

## Background

On April 14^th^, 2016 the British Columbia (BC) Provincial Health Officer declared a public health emergency in response to a dramatic rise in drug overdoses [1]. Peer workers, i.e., individuals with past or present substance use experience who use that lived experience to inform their professional work [2–4], are at the forefront of overdose response in BC [5–7]. Peer workers perform a variety of roles, including responding to overdoses, distribution of naloxone and other harm reduction supplies, peer witnessing of drug use, referrals to services such as housing agencies, advocacy, outreach, and research [8]. Employing peers in overdose response settings is critical in increasing the accessibility and acceptability of programs for people who use substances [4, 9, 10]. There is an increasing body of knowledge that supports the impact of peer work on reducing harms associated with drug use and structural violence [3, 4, 11–13]. The onset of the Coronavirus Disease of 2019 (COVID-19) pandemic is correlated with escalating rates of overdoses and increased work and stress for peer workers due to closures and reduced hours of organizations providing services to people who use drugs (PWUD) [14].

Although peer work has multiple benefits for service users and employees alike, the work is stressful, with lasting emotional and mental health effects [4, 13, 15–18]. Unlike other healthcare providers and first responders [19, 20], peer workers usually lack access to occupational and mental health supports. Even within organizations that employ peer workers, staff without lived/ living experience have access to more workplace supports [21]. Very few resources or programs are available for people with lived/living experience of substance use [22–25], and this can lead to compassion fatigue and burnout [26–28]. This lack of supports, workplace discrimination and stigma are situated more broadly within a legal framework that criminalizes drug use [29, 30], placing peer workers at a disadvantage in terms of income, education and housing. There is a critical need for supports for peer workers that recognize their unique positioning as people with lived/living experience within a professional setting as well as the often-adverse effects of working in overdose response environments.

Intervention Mapping (IM) creates theory- and evidence-based health promotion programs via community-based development processes to ensure that the intervention adequately addresses community needs [31, 32]. As such, we identified IM as an appropriate framework to use within the Peer2Peer Project, which aims to identify, implement and evaluate peer-led interventions that are feasible and effective in supporting peer workers in overdose response settings in BC [33]. IM is informed by socio-ecological theory and prioritizes multi-level intervention planning by evaluating individual, inter-personal, organizational, community and societal influences on health outcomes [31, 32]. In this paper, we describe in detail how a support intervention for peer workers, titled ‘ROSE’, was conceptualized. We conclude with recommendations for other organizations employing peer workers to tailor the intervention components for their respective settings.

## Methods

**Fig. 1:**
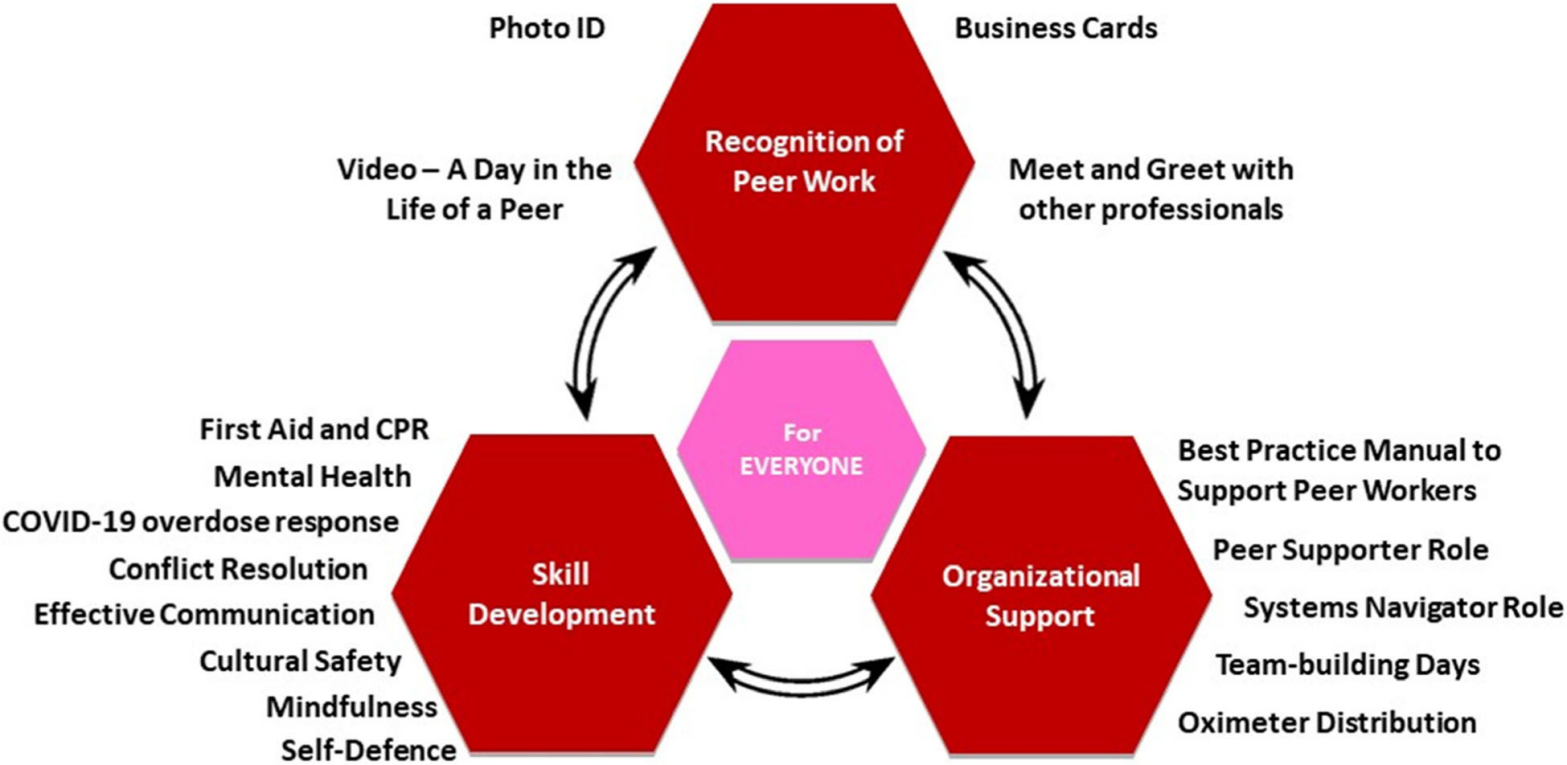
Components of the ROSE model and the strategies within each component

The ROSE model (Fig. 1, described later) was developed through a community-based research project conducted in collaboration with two organizations in BC. The first, SOLID Outreach Society, is a peer-led organization in Victoria, BC that educates, advocates and provides services for individuals that use substances [34]. Staff at SOLID Outreach Society are individuals with lived/living experience of substance use (peer workers). The second pilot organization for this project is RainCity Housing - a not-for-profit, housing-first organization in Vancouver Coastal and Fraser regions that provides housing and support services for people living with mental health, substance use, and other challenges [35]. For this project, we worked with RainCity Housing sites in Vancouver, Maple Ridge and Coquitlam. RainCity employs multi-disciplinary staff with and without lived/living experience of substance use. These include social workers, administrative staff, outreach workers, healthcare professionals, and peer workers. Peer workers at both organizations are involved in a variety of tasks including outreach and community building, responding to overdoses, distribution of naloxone and other harm reduction supplies, “Rig Dig” which is the collection and safe disposal of used needles and trash, peer witnessing of drug use, peer-to-peer support and debriefing, and overall contribution to the operations of the facility by assisting with and maintaining a safe, clean and welcoming program space. The target population for our study is all peer workers at both these organizations.

The project team consists of 12 team members; two principal investigators, one project manager, one project assistant, and eight peer research assistants (PRAs) who are people with lived/living experience of substance use, selected by each pilot site to join the team and represent the voices of other peer workers within their organizations. As such, all PRAs had robust and trusted relationships with individuals at each organization. Further, all PRAs were trained in qualitative research practices and ethics. The team consists of a diversity of ages, sexes, and educational backgrounds.

### Designing the model

The Intervention Mapping (IM) protocol developed by Bartholomew et al. (2016) follows a structured approach guided by a six-step protocol which describes the iterative path from problem identification to evaluation of the intervention [32]. Each of the six steps of IM comprises several tasks which integrate theory and evidence [32].

To design the ROSE model, we followed each of the six steps outlined by IM. These steps include: needs assessment, specification of program objectives, selection of theory-based methods and strategies for addressing identified needs, creating a program plan, creating an implementation plan and creating an evaluation plan [31, 32]. Below we describe application of the steps of IM to design the ROSE model. Activities and outcomes taken at each step of IM are described within the results section. For Steps 5 and 6, we outline the proposed plans, but the results of these steps will be reported elsewhere.

## Results

### Step 1: Needs Assessment

Eight focus groups with 31 participants in total were conducted between November 2018 and March 2019; two in the Fraser region (Maple Ridge and Coquitlam), two in Vancouver, and four in Victoria. Details on recruitment of participants, interview processes, and data analysis are described in two previously-published articles [21, 36].

All focus groups were facilitated by PRAs, with an academic researcher present to take field notes. Focus group discussions were guided by a facilitator guide developed for this study and piloted by the research team (see Supplementary File 1). Focus group participants provided consent and were given the option to complete a demographic questionnaire, provided as a Supplementary File 2. The consent form included a description of the project goals, the biography of the key researchers, and their contact information.

The participants were diverse; just over half were male (55%). Of those that completed the questions related to age and education (*n*=18, 58% of total), 56% were over the age of 40 and all reported having received at least some high school education. The demographic profile of these participants is presented in Table 1.

**Table 1:** Demographic Characteristics of Focus Group participants (*N*=31)

**Sex**	**%**
Male	55%
Female	45%
**Age**	**%**
Under 20	0%
21- 30	6%
31- 40	19%
41- 50	13%
51-60	6%
61-70	10%
71+	3%
Unknown	42%
**Highest Level of Education**	**%**
Some high school	23%
Completed high school	10%
Some community college or technical school	6%
Completed comm. college or technical school	6%
Some university	3%
Completed Bachelor’s degree	6%
Post Graduate Training	3%
Unknown	42%

Using a participatory coding process described in previously-published articles [21, 36], the project team identified multiple issues faced by peer workers. Using interpretive description to delve deeper into the quotes and situate the issues within a real-world context [37], the project team categorized the issues according to levels of the Socio-Ecological Model (SEM), “a framework for understanding the multiple levels of a social system and interactions between individuals and environment within this system” [38]. The master list of issues faced by peer workers sorted into different SEM levels is presented in Table 2. The peer workers prioritized certain issues that they hoped to tackle through the intervention (Table 3). These priority areas were classified into three overarching themes; Recognition of Peer Work, Organizational Support and Skill Development.

**Table 2:** Master list of issues identified by peer workers from a socio-ecological perspective

Socio-Ecological Model Level	Issues faced by peer workers
Individual	• Lack of adequate skills and confidence. Examples of topics identified include recognizing signs and symptoms of mental health disorders and challenges, self-defence, conflict resolution, first aid, and communication. • Personal health and lack of opportunities for self-care and de-stressing.
Inter-personal	• Lack of access to peer-to-peer debriefing. • Strained relationships with other professionals e.g. Police and paramedics.
Organizational	• Inequity in the workplace including inequitable pay, differential treatment of peer workers. • Precarious/ unstable jobs. • Lack of respect and recognition at work. • Lack of role clarity.
Community	• Lack of respect and recognition for peer workers in the community (by other professionals). • Lack of access to and awareness of community resources such as government issued ID cards, detox, etc.
Policy or Society	• Stigma. • Low minimum wage. • Harmful policies such as criminalization of drug use. • Lack of adequate housing supports.

**Table 3:** Interventions proposed for key issues identified and prioritized

Component	Key Issues	Strategies Developed
**Recognition of Peer Work**	• Peer workers felt that they do not get the respect they deserve from other professionals. • Peer workers face considerable amount of stigma from the general public which hinders their access to services. • Peer workers are not taken seriously at work and pushed aside when “professionals” such as paramedics arrive, especially at the scene of an overdose.	• Organize meet and greet events between peer workers and other professionals such as police and paramedics to facilitate relationship-building. • Create a video featuring a day in the life of a peer worker to create awareness about the work done by peer workers. • Introduce photo IDs and business cards as symbols of professionalism and to increase legitimacy for peer workers’ roles.
**Organizational Support**	• There is lack of clarity around the role of peer workers and the word “peer” is stigmatizing. • Peer workers are paid less than other staff without lived/ living experience of substance use. • Lack of clarity around expectations at work. • Lack of effective communication between peer workers and staff without lived/ living experience. • Lack of opportunities for peer workers to unwind, debrief or de-stress despite the stressful nature of their work. • Unstable living conditions and lack of access to external resources which affect peer workers’ productivity and mental health.	• Create job descriptions with a formal job title. • Indicate recommended pay on the job description based on BC peer payment standards [22]. • Create a contract highlighting expectations at work, scheduling guidelines, break policies, etc. • Introduce team-building days to foster relationship-building and improved communication. • Introduce a Peer Supporter role at each site so peer workers have someone to reach out to for debriefing and support. • Introduce a Systems Navigator role at each site to provide referrals for external services including housing.
**Skill Development**	Peer workers identified several skills they would like to develop further for increased competency and self-confidence. These include: First Aid and CPR, overdose response techniques, self-defence, communication skills, mental health awareness, cultural competency, conflict resolution, self-care, etc.	Introduce training for peer workers covering the topics identified by them as priorities. For topics where existing training was adequate, in-person sessions were organized. For topics that did not have existing trainings for peer workers, online training modules were developed with input from the team.

Within the ‘recognition’ theme, one of the top issues encountered by peer workers is that they lacked respect from their work colleagues and other professionals they encounter in their work. This dynamic manifests through inequity in the workplace, lack of basic workplace resources, and strained relationships with other service providers [21].

The issues identified under ‘organizational support’ include lack of organizational and mental health supports for peer workers. Lack of such resources often led to low morale and burnout [21]. As highlighted in another paper, many peer workers indicated a lack of job clarity and formalized contracts with their organizations, leading to poor working conditions and relegation to menial labour by supervisors and co-workers [21]. Inequitable pay, despite the similar nature of work done by support workers without lived/living experience, was another issue.

Peer workers also identified skill development as a need and suggested topics that would help increase their self-confidence and capacity. Identified training needs included technical skills, people skills and self-care skills. Technical skills identified included first aid and CPR, recognition of signs and symptoms of mental health disorders, naloxone administration and use of pulse oximeters. Under people skills, peer workers identified the need for training in conflict resolution and de-escalation, communication skills, peer debriefing skills and cultural safety. Self-care skills included mindfulness and self-defence.

### Step 2: Specification of Program Objectives

The three themes describing the support needs of peer workers outlined in Step 1 formed the basis of the intervention model, from which the team collaboratively came up with a list of potential names. Through an anonymous voting process, the team named the intervention model ‘ROSE’, with each letter standing for one of the three themes (recognition, organizational support, skill development). The E in the ROSE model emphasizes that the resources developed are for Everyone, and highlights its inclusivity and the commitment of the peer workers at the pilot sites to make the resources available to all organizations across BC (see Fig. 1).

The overall aim of the ROSE model is to increase support for peer workers in overdose response settings, enabling them to stay motivated and work optimally in a stressful work setting, with reduced emotional, mental, and social stress. The objectives of the ROSE model are to: 1) Facilitate equitable access to workplace resources for peer workers, 2) Provide training and education for peer workers to improve their skills and gain self-confidence, and 3) Increase awareness and recognition among individuals without lived/living experience about the crucial work done by peer workers in overdose response settings.

Through these objectives, the ROSE model aims to facilitate culture change within organizations, leading towards more equitable and just workplaces. This, in turn, will lead to a positive impact at various socio-ecological levels, including improved self-confidence of peer workers at the individual level, formation of social networks and relationships with colleagues and other professionals at the interpersonal and community levels, more equitable and just workplaces at the organizational level, and ultimately a more accepting and less stigmatizing society.

Each component of the ROSE model consisted of several strategies informed by theory, evidence, and the lived/living experience of PRAs (Fig. 1). Details of the strategies within each component are specified below.

### Step 3: Selection of theory-based methods and strategies for addressing identified needs

Having specified the objectives and priorities of the intervention, we selected a range of theories to guide the selection and design of strategies. We specifically considered the Ottawa Charter for Health Promotion [39] which includes strengthening community action, development of personal skills, and creating supporting environments.

Given our focus on organizational-level interventions, the Organizational Development Theory was chosen as the primary theory for intervention planning. This theory is defined as “a system-wide process of applying behavioural science knowledge to the planned change and development of the strategies, design components, and processes that enable organizations to be effective” [40]. Specifically, we focused on creating an intervention that would affect organizational culture, i.e. assumptions and beliefs which govern the behaviour of members of the organization, and organizational climate, i.e. the personality of the organization [40].

### Step 4: Creating a Program Plan

For each of the issues identified and prioritized by peer workers, an intervention strategy was decided upon during the bi-weekly meetings with the project team. This step was informed by evidence from literature and by peer worker suggestions based on their lived/ living experience. This led to the creation of a comprehensive yet feasible intervention model to address the identified needs of peer workers in overdose response settings. Strategies within each component of the ROSE model are described below and summarized in Table 3 and Fig. 1.

#### Recognition of Peer Work

Three primary strategies were identified and implemented under the “Recognition” component of the ROSE model. First, peer workers identified the need for basic resources such as photo identity cards (ID cards) and business cards as tangible symbols of professionalism, authority and validity within their roles. Pilot sites were provided with portable ID card printers and business card templates so that all peer workers employed could receive their individual photo IDs and business cards.

Secondly, to create awareness about the work done by peer workers among the general public, including among other professionals that peers work with, a video titled #PeerLife, featuring a day in the life of a peer worker, was developed [41]. This video featured the story of four peer workers, one from each of the pilot sites, and their day-to-day work in the face of the overdose crisis. The video highlights the harsh realities faced by peer workers and encourages recognition and appreciation for their work. This video is available on YouTube, has been promoted through social media, and featured on the Toward the Heart website [42].

A third recommended strategy was meet and greet events between peer workers and other professionals including police and paramedics. The purpose of these events was to foster relationship-building and to raise awareness about the crucial roles fulfilled by peer workers. However, due to COVID-19 restrictions, we were unable to implement and evaluate this particular strategy at the pilot sites.

The strategies under the “Recognition” component span multiple levels of the socio-ecological model. Provision of photo IDs and business cards to peer workers, for example, constitute organizational-level interventions, while meet and greet events foster inter-personal relationships between peer workers and other professionals. These relationships, in turn, can help to improve peer workers’ work experience (organizational-level) and may address negative attitudes and stigma towards peer workers (societal-level). Similarly, the #PeerLife video, which creates awareness about the role of peer workers among the general public is a societal-level intervention since it is a first step towards addressing stigma and negative attitudes towards PWUD (see Table 2).

#### Organizational Support

The “Organizational Support” component of the ROSE model consists of several strategies. To create role clarity, formal job descriptions were created, which solidified the role of peer workers and suggested a living wage based on BC’s peer worker pay standards [22, 43]. Formal employment contracts which detailed the terms and expectations of employment were developed. These documents were implemented at the pilot sites and templates of these documents were compiled into a Best Practice Manual to Support Peer Workers and made publicly available for other organizations to adapt based on their needs [44].

Additionally, two roles were created at SOLID Outreach Society: Peer Supporter and Systems Navigator. For the Peer Supporter role, a person with lived/living experience of substance use was hired to provide peer-to-peer debriefing. The rationale for this role was the knowledge that shared experience helps to facilitate trust, understanding and a special bond of care and comfort [36]. 

The second role was that of a Systems Navigator whereby a person with lived/ living experience of substance use was hired to support peer workers in navigating access to external services. These include assisting peers with access to harm reduction services, accompanying peer workers to healthcare visits, providing legal support, supporting peer workers to apply for their government identification cards, providing assistance to complete housing applications and income assistance forms, providing referrals and reference letters for housing applications, and referring peer workers to detox or treatment, if desired. The Systems Navigator also builds relationships with external service providers and is acquainted with systems and services to provide easy referrals to peer workers. As such, the Systems Navigator can assist in enabling access to external resources which are most often institutionally inaccessible for people who use substances.

Teambuilding days were also organized to boost peer workers’ morale and provide them an opportunity to de-stress and unwind. For each pilot site, it was proposed that teambuilding days be organized twice a year with an emphasis on fun activities such as bowling and holiday parties. However, due to COVID-19, not all activities were organized, as planned. Other gestures, such as provision of gift cards and thank you notes, were implemented to improve morale.

In addition to addressing the needs of the peer workers identified during the focus groups, the “Organizational Support” component of the intervention also included resources identified during the bi-weekly check-in and progress meetings with the team. One such resource was the need for pulse oximeters in response to increasing reports in BC of substances containing mixtures of opioids and benzodiazepines and the identification of unregulated etizolam in urine drug screens [45–47], causing people to remain unconscious even after naloxone was administered and breathing restored [45–47]. Pulse oximeters aid in identifying when oxygen levels are within normal range and rescue breaths are not needed, which is of particular importance since the onset of COVID-19.

Like strategies in the “Recognition” component, the “Organizational Support” strategies span multiple levels of the socio-ecological model. For example, the Peer Supporter role is an organizational-level intervention, however, peer workers may realize improved mental health through engagement with the Supporter, and this is an individual-level factor. Similarly, the hiring of a Systems Navigator is done at the organizational level, but through relationship-building and referrals, this role's aim is to increase peer workers’ access to external community resources, thus also operating at the community level. Teambuilding days help to improve relationships between colleagues (interpersonal) and boost morale and motivation (individual). Through the implementation of organizational supports, the ROSE model has the potential to challenge the norms and address the negative attitudes and stigma towards peer workers both within the organization (organizational) and in society in general (societal) (See Table 2).

#### Skill Development

Training sessions and resources were organized and/or created based on identified need. For some topics, such as first aid, well-recognized external training already existed. In such cases, peer workers were supported to attend these external trainings and earn certification. In addition to providing peer worker training on topics identified during the focus groups, several other training materials were developed to meet the situational needs of peer workers. For example, , information sheets and training videos on responding to overdoses in light of COVID-19 were developed.

For topics where there was a lack of existing training tailored for peer workers, the Peer2Peer team developed a standardized BC peer worker curriculum, which was developed by and for peer workers. This training curriculum consists of five modules: mental health awareness, effective communication, conflict resolution, peer-to-peer counselling and debriefing skills, and organizational etiquette. It is critical that peer worker training or capacity-building be tailored to the realities of people who use substances as many workplace training programs are designed for people without lived/living experience of criminalization or other impacts of drug use.

#### Step 5: Creating an Implementation Plan

Next, the project team defined the scope of the intervention and decided on the project activities for each pilot site. The sequence of activities was determined, prioritizing the strategies that were easiest to implement and had potentially high impact. PRAs and representatives from each partner site decided on the start and end dates for each activity, milestones, budget, and task assignment, which was documented as detailed site-specific action plans.

To guide the implementation of the interventions, the Normalization Process Theory (NPT) was utilized. NPT is a mid-range implementation theory that explores how components of an intervention are implemented, routinely embedded in everyday practice, and integrated within organizational settings [48]. Consistent with NPT, we considered four theoretical constructs: (1) coherence (how people understand the practice) (2) cognitive participation (how people engage in a practice); (3) collective action (how the practice interacts with existing practices) and; (4) reflexive monitoring (how a practice is understood) [48]. NPT encompasses both the implementation process as well as the outcomes of the intervention [48].

NPT has been applied to numerous health studies and helps to guide and explain implementation of new processes [49]. As such, it was an appropriate theory to inform the ROSE model.

#### Step 6: Creating an Evaluation Plan

The final step of intervention mapping was to create the evaluation plan. Consistent with NPT, we developed both a process and outcome evaluation plan.

To assess the process of implementing several aspects of the ROSE model, we conducted qualitative interviews with both implementors and recipients of the intervention, i.e., the organizational managers and peer workers. This evaluation was informed by NPT and conducted in collaboration with the partner organizations.

To assess the outcomes of the interventions, a survey informed by the focus group findings was conducted. This survey consisted of demographic questions, measures of peer workers’ perceptions of health and quality of life, substance use patterns and working conditions. The majority of the survey questions were adapted from validated tools with good psychometric properties, including the Canadian Community Health Survey [50], Short Form – 12 (SF-12) Health survey [51], the Professional Quality of Life Scale (ProQOL) [52], and the Job Satisfaction Survey [53]. The survey was conducted both prior to implementation of the interventions as well as one year after implementation in order to assess the change in these measures attributed to the interventions.

## Discussion

As highlighted in our previous paper [21], peer workers face multiple sources of stress both in their personal lives and at work. The issues faced by peer workers span multiple levels of the socio-ecological model, calling for multi-levelled interventions. The ROSE model is an organization-level intervention that can potentially have impacts across multiple levels.

A review of workplace interventions indicates that the majority of workplace interventions aim to reduce work-related stress and take one of three forms: primary, which are typically for all employees and have a focus on prevention, secondary which target employees who have been exposed to risk factors and provide employees with knowledge and skills to cope with the stressor, and tertiary which focus on employees who are experiencing distress and need assistance in recovering from stress-related symptoms [54]. Interventions in these three forms are limited in that they tend to focus on individuals rather than on organizations. The ROSE model does contain a mixture of primary, secondary and tertiary strategies, but goes beyond the individual to include organizational level changes and a focus on organizational culture change. Organizational changes are more likely to be sustained as they are embedded in everyday practices within organizations.

In designing the ROSE model, the Peer2Peer team also considered the success of similar interventions in different contexts. For example, past studies have indicated that emotionally-supportive exchanges between peers can foster feelings of being accepted, cared for, empathized, respected, and valued despite profound personal difficulties [55–57]. This evidence informed the creation of the role of a Peer Supporter within the “Organizational Support” component of the intervention. Furthermore, according to a review of interventions for reducing stigma, educational interventions can help to improve attitudes of individuals without lived/living experience towards PWUD [58]. As such, many of the interventions in the “Recognition” component of the ROSE model are based upon the premise of creating awareness of peer work among the general public, including other professionals that peers work with, to address negative attitudes towards them. There was also support in this review for interventions which engage PWUD as relationship-building with people with lived/living experience of substance use can address social and structural stigma [58]. This informed the implementation of meet and greet events between peer workers and service providers they often encounter, such as police officers.

The strategies within each component of the ROSE model are strongly aligned with the organizational development theory as it aspires to positively change the organizational culture and climate. The ROSE model has the potential to facilitate relationship-building between peer workers and other professionals, instilling positive attitudes and behaviours that may translate into a way of life over time [59, 60]. In this way, the ROSE model can have organization- and community- wide ripple effects, allowing all the staff at the organization to value an inclusive and just workplace. The implementation of the ROSE model is also aligned with NPT because it is aimed at not only instilling a temporary change in behaviour and attitudes within an organization, but hopes to achieve long-term effects which become routinely embedded in everyday practice and integrated within organizational settings [48].

One of the strengths of the ROSE model is that it was informed by a diversity of participants, i.e., the participants during the focus groups (needs assessment) were from different age groups, sexes, and locations. This provided a range of views on the support needs of peer workers and potential solutions to meet the needs. Second, each of the strategies that were decided upon were strongly informed by theory and evidence from literature, which increases the chances of success. In addition, and most importantly, the intervention was shaped by the lived/living experience of PWUD and centred the voices and opinions of peer workers. Engaging peer workers ensures that the strategies implemented are not only relevant but also acceptable for peer workers at the partner sites, and beyond. By centring peer workers in intervention planning and implementation, the ROSE model challenges the culture of oppression rooted in the colonial endeavour to make decisions *for* marginalized populations rather than *with* them.

A potential limitation of the model is that it is based on needs assessment data from focus groups whereby participants may have been hesitant to express their opinions due to fear of judgement from other participants. In an attempt to mitigate this issue, focus groups were kept small and were facilitated by PRAs to allow for maximum participation and to promote a power balance. Another limitation of the intervention is that the partner sites were located within four metropolitan or large urban centers in BC and we lacked the insights of peer workers in rural settings. The partner sites, however, do represent diversity in type i.e., housing agency versus non-housing, and geography i.e., four different cities. More research is warranted to test the applicability of the intervention for peer workers in other contexts in BC and in Canada. Lastly, the authors recognize that although interventions within the ROSE model are informed by theory and evidence, their ability to impact societal level factors such as stigma is minimal. This stigma stems from a history of drug prohibition in Canada [29, 30], leading to inequity in income, education and housing for PWUD. Unless measures to address these underlying issues are taken, such as decriminalization, peer workers may never fully feel supported. Yet, the ROSE model is an organizational intervention with potential ripples and impacts at multiple levels in the socio-ecological context.

## Conclusion

Using the IM process, we developed the ROSE model which consists of three components (Recognition of peer work, Organizational support and Skill development for Everyone). Each component includes a set of strategies designed to meet the specific objectives of the intervention. The development of the ROSE model was rooted in the lived and living experience of peer workers and was informed by previous literature and health promotion theories. Importantly, the ROSE model is an organizational level intervention which has the potential for impact at individual, inter-personal, organizational, community and systems levels and requires further evaluation to determine its efficacy.

## Supplementary Information

**Additional file 1.**

**Additional file 2.**

## Data Availability

The datasets used and/or analyzed during the current study are not publicly available due to the small nature of peer networks and potential for identification, but are available from the corresponding author on reasonable request.

## Abbreviations

BC: British Columbia
BCCDC: British Columbia Centre for Disease Control
COVID-19: Coronavirus Disease of 2019
IM: Intervention mapping
PRA: Peer research assistant
PWUD: People who use drugs
ROSE: Recognition of peer work, Organizational support and Skill development for Everyone
