# Supplementary material for: Using intervention mapping to develop ‘ROSE’: an intervention to support peer workers in overdose response settings
Source: BMC Health Serv Res. 2021 Nov 27;21:1279. doi: 10.1186/s12913-021-07241-2 (PMC8626711; doi:10.1186/s12913-021-07241-2)
Supplement: Supplementary file 1 — Additional file 1. [file 12913_2021_7241_MOESM1_ESM.docx]

**P2P FOCUS GROUP GUIDE**

***INTERVIEWER***: State location and date recorder.

*These questions are a guide.*

**Engagement Questions**

- To start, can you tell me about your work re Overdose Response Environment?
- What do you like about overdose work? What supports you in your work?
- What are some of the challenges in overdose work? What do you find stressful?
- How do you handle stressful situations at work?
- How do you take care of yourself after work?

**Exploration Questions**

***INTERVIEWER:*** This research project is to develop and implement peer led strategies to support Peers working in Overdose Response environments. SOLID and Rain City peers came up with some beginning ideas about what is important to peer support? We want to know what you think of these ideas. One of the first things that the advisory group talked about was the need to improve health and wellbeing. There are different ways to do this. We’re going to go through a few different examples, and we’re hoping you can tell us your thoughts on each example.

*Interviewer: hand out model (attached) to focus group questions. Before we go in depth …...*

- What are your first impressions?
- Anything obvious missing? What would you change or add?

Now, let’s go through each part of the model…

**Peer-to-Peer Debriefing or Counselling**. The idea is that there would be training that would provide skills that would allow for Peer workers to help each other after tough days. What do you think of this idea? Would it be helpful to you or your co-workers? If so, what should it look like?

**Creative art and music therapy.** This would look like time set aside for you to express yourself through art or music. What do you think of this idea? Would it be helpful to you or your co-workers? If so, what should it look like?

**Assistance with living condition:**  What do you think about having a designated person or advocate that could help with living conditions and accessing other resources?

What do you think of this idea? Would it be helpful to you or your co-workers? If so, what should it look like?

W**ork place supports** – things that are at your work that help you in your day-to-day work. It could be having a break room where you’re able to go to during your workday or having someone you could go to if you needed some extra help during the day. What do you think of this idea? Would it be helpful? If so what would it look like where you work?

**Skill development and training** is important.

- - What do you think of this idea?
  - What sort of training do you think is needed?
  - What would the training process look like?
  - What do you think training should include? (*Probes:* *mindfulness, communication and advocacy skills*).

**Equitable Pay**: We’ve also heard that some Peers working in Overdose Response Environment feel that their work is not appropriately compensated and valued.

- - How do you feel about this?
  - Something else we talked about was how equitable pay and working conditions is tied to overall health and well-being – do you agree or disagree?

**Exit Questions**

- Is there anything else that you would like to say about your work?
- Is there anything else that you would like to say about supports or programming that are needed?
- Is there anything else that we haven’t spoken about that you think we should focus on?
