# Supplementary material for: Using intervention mapping to develop ‘ROSE’: an intervention to support peer workers in overdose response settings
Source: BMC Health Serv Res. 2021 Nov 27;21:1279. doi: 10.1186/s12913-021-07241-2 (PMC8626711; doi:10.1186/s12913-021-07241-2)
Supplement: Supplementary file 2 — Additional file 2. [file 12913_2021_7241_MOESM2_ESM.docx]

**DEMOGRAPHICS: FOCUS GROUPS**

Date: _____________________________

Interviewer: _________________

Location: __________________

1) What is your age? □□ years

2) What gender do you identify as?

- Female
- Male
- Transgender
- OR ________________ (Please specify)

3) What is the highest level of education you have completed?

*(check ONE box only)*

- No schooling
- Some elementary schooling
- Completed elementary school
- Some high school
- Completed high school
- Some community college
- Some technical school (college classique CEGEP)
- Completed community college
- Completed technical school (college classique CEGEP)
- Some university
- Completed Bachelor’s Degree
- Post graduate training: MA, MSc., MSW
- Post graduate training: PhD, “Doctorate”
- Professional degree (Law, Medicine, Dentistry)
- Don’t know
- Prefer not to say

4) Years of experience as a Peer Worker? □□ years

5) Length of time doing overdose prevention? □□ years

6) Location of where you do overdose work?

- housing
- shelter
- outreach
- drop in program
- other ___________________
